# Renal Lesions

**Published:** 1889-12-07

**Authors:** 


### RENAL LESIONS.

Renal lesions appearing after a febrile attack of doubtful nature are commonly and justly considered as presumptive of scarlatina or of diphtheria, but Dr. Fliessinger, in a recent epidemic of that really rare and peculiar disease, influenza, found in a number of cases lesions identical with those met with in scarlatina; not only renal congestion without dropsy, but a few examples of well-marked acute desquamative nephritis. All, however, recovered in two or three weeks after having been kept in bed on a milk diet.

